# Adenovirus-Mediated CCR7 and BTLA Overexpression Enhances Immune Tolerance and Migration in Immature Dendritic Cells

**DOI:** 10.1155/2017/3519745

**Published:** 2017-03-14

**Authors:** Haiming Xin, Jinhong Zhu, Hongcheng Miao, Zhenyu Gong, Xiaochen Jiang, Xiaoyan Feng, Yalin Tong

**Affiliations:** The Burn and Plastic Surgery Department, PLA 181 Hospital, Guilin, Guangxi, China

## Abstract

Our previous report revealed that immature dendritic cells (imDCs) with adenovirus-mediated CCR7 overexpression acquired an enhanced migratory ability but also exhibited the lower immune tolerance observed in more mature cells. In the present study, we aimed to investigate whether BTLA overexpression was sufficient to preserve immune tolerance in imDCs with exogenous CCR7 overexpression. Scanning electron microscopy and surface antigens analysis revealed that BTLA overexpression suppressed DC maturation, an effect further potentiated in CCR7 and BTLA cooverexpressing cells. Correspondingly, in vitro chemotaxis assays and mixed lymphocyte reactions demonstrated increased migratory potential and immune tolerance in CCR7 and BTLA coexpressing cells. Furthermore, CCR7 and BTLA cooverexpressed imDCs suppressed IFN-*γ* and IL-17 expression and promoted IL-4 and TGF-beta expression of lymphocyte, indicating an increase of T helper 2 (Th2) regulatory T cell (Treg). Thus, these data indicate that CCR7 and BTLA cooverexpression imparts an intermediate immune phenotype in imDCs when compared to that in CCR7- or BTLA-expressing counterparts that show a more immunocompetent or immunotolerant phenotype, respectively. All these results indicated that adenovirus-mediated CCR7 and BTLA overexpression could enhance immune tolerance and migration of imDCs. Our study provides a basis for further studies on imDCs in immune tolerance, with the goal of developing effective cellular immunotherapies for transplant recipients.

## 1. Introduction

Allogenic skin grafts are an ideal way to treat patients with severe burns; however, immune rejection usually results in a complete failure of the transplanted tissue. Immunosuppressive drugs are currently used to prevent graft rejection, but these are often accompanied by strong adverse side effects resulting from diminished immune function [1–3]. As such, therapeutic approaches that facilitate immune tolerance and simultaneously preserve immunocompetence in transplant patients urgently need to be developed.

Dendritic cells (DCs) are the most potent antigen-presenting cells in the body [4]. Previous studies have shown that DCs can initiate either immunogenic or tolerogenic pathways depending on their maturational state and location [4]. It is generally believed that immature DCs (imDCs) modulate tolerance, whereas mature DCs facilitate T cell activity and inflammation [5, 6]. Significantly, recent studies have found that imDCs can induce immune tolerance and limit transplant rejection in a variety of organ grafting experiments, including tooth [7], renal [8], corneal [9], and skin allografts [10]; however, these effects were modest and required a large number of cells, stemming from their weak lymph-node homing ability.

C-C motif chemokine receptor 7 (CCR7) is crucial for DC and T cell lymph-node homing [11] and regulates DC entry into the lymphatic capillaries of peripheral organs, as well as the extravasation of LN-resident DC subsets across high endothelial venules (HEVs) [12]. Our group previously reported that imDCs with adenovirus-mediated CCR7 overexpression acquired an enhanced migratory ability, but also exhibited the lower immune tolerance observed in more mature cells [13].

Negative regulators of T cell activation have been identified as targets to design novel strategies aimed at prolonging graft survival and promoting immunological tolerance and translated to the clinic [14]. B and T lymphocyte attenuator (BTLA) is a new member of negative regulators of T cell activation that suppresses T cell activation through its interaction with herpesvirus entry mediator (HVEM), a member of the tumor necrosis factor receptor superfamily [15, 16]. It is explicit that BTLA is required for DCs to actively adjust tolerizing T cell responses under steady-state conditions [17]. In the present study, we aimed to investigate whether BTLA overexpression was sufficient to preserve immune tolerance in imDCs with exogenous CCR7 overexpression.

## 2. Materials and Methods

### 2.1. Generation of imDCs from Mouse Bone Marrow-Derived Mononuclear Cells

C57BL/6 mice were bred, maintained, and used in accordance with the protocols established by the ethics committee on animal use in experimental animal facilities of Number 181 Hospital of PLA. Bone marrow-derived imDCs were generated as previously described, with some modifications [18]. Briefly, bone marrow cells were harvested by flushing the femurs and tibias of 6–8-week-old female mice with medium under aseptic conditions. After the separation of erythrocytes, the harvested marrow was cultured in complete RPMI with 10% fetal bovine serum (FBS). On day 2, the culture medium was replaced with fresh RPMI supplemented with 10% FBS, 100 ng/mL granulocyte monocyte colony-stimulating factor (GM-CSF), and 50 ng/mL IL-4 (PreproTech, Rocky Hill, NJ, USA). Half of the medium was replaced with fresh medium and cytokines every 2 days. On day 5, nonadherent cells were used as imDCs for subsequent adenoviral infection.

### 2.2. Plasmid Construction and Generation of Recombinant Adenovirus

The complete CCR7 open reading frame (ORF) was PCR-amplified with primers containing ApaI or NotI restriction sites (5′-agggggcccgccaccATGGACCCAGGGAAACCCAGG-3′ and 5′-ataagaatgcggccgcCTACGGGGAGAAGGTTGTGGTG-3′, resp.) and inserted into the pDC316-mCMV-EGFP shuttle vector to generate pDC316-mCMV-CCR7-EGFP. Similarly, the BTLA ORF was amplified with primers containing NotI or HindIII restriction sites (5′-ataagaatgcggccgcgccaccATGAAGACAGTGCCTGCCATGCTTG-3′ and 5′-cccaagcttTTAACTTCTCACACAAATGGATGCATA-3′-, resp.) and inserted into the pDC316-mCMV-tdTomato shuttle vector to generate pDC316-mCMV-BTLA-tdTomato. Viral particles were produced by cotransfecting 293 cells with pDC316-mCMV-CCR7-EGFP or pDC316-mCMV-BTLA-tdTomato and the adenovirus genomic plasmid pBHGloxΔE1, 3Cre, using Lipofectamine 2000 (Promega, Madison, WI, USA). Transfected cells were incubated for 7 days at 37°C and then lysed with three consecutive freeze/thaw cycles. Crude recombinant virus was collected from the supernatant by centrifugation and subjected to three rounds of plaque purification in 293 cells. CCR7 and BTLA adenoviral titers were then determined by qRT-PCR.

### 2.3. Cell Samples Preparation

The generated imDCs were divided into 4 groups: group 1: imDCs infected with adenovirus expressing EGFP only (Ad.EGFP); group 2: imDCs infected with adenovirus expressing CCR7 and EGFP (Ad.CCR7); group 3: imDCs infected with adenovirus expressing BTLA and tdTomato (Ad.BTLA); group 4: imDCs infected with both adenovirus expressing CCR7 and EGFP and adenovirus expressing BTLA and tdTomato (Ad.CCR7 + BTLA).

### 2.4. RNA Extraction and Quantitative Reverse Transcription PCR (qRT-PCR)

Total RNA was extracted from cells with TRIzol reagent (Invitrogen, Carlsbad, CA, USA) and 1 *μ*g was reverse transcribed into cDNA, using M-MLV Reverse Transcriptase (Invitrogen) in a 20-*μ*L reaction, according to the manufacturer's protocols. CCR7, BTLA, IL-10, and HVEM gene expression were then analyzed by qRT-PCR in a ABI PRISM 7500 Sequence Detection System, using SYBR Green qPCR SuperMix (Invitrogen). Glyceraldehyde-3-phosphate dehydrogenase (GAPDH) expression was used as an internal control. Primers for qRT-PCR were as follows: CCR7, forward: 5′-CTTTCTTGTATGCCTTCATC-3′ and reverse: 5′-GGTTAAGCAGTTTCTTAGGT-3′; BTLA, forward: 5′-GTGAATAAAGAGGCCTTACT-3′ and reverse: 5′-CCTGAACAAGCTTAACTAGA-3′; IL-10, forward: 5′-CAAGCCTTATCGGAAATGAT-3′ and reverse: 5′-TAGAGAGCTCTGTCTAGGTC-3′; HVEM, forward: 5′-TATGTGCTGACTGCCTAACA-3′ and reverse: 5′-CGTCTTACTTCCTGTTGAAATG-3′; GAPDH, forward: 5′-GGCCTCCAAGGAGTAAGAAA-3′ and reverse: 5′-GCCCCTCCTGTTATTATGG-3′. Experiments were performed in duplicate and repeated three times. Fold induction of gene expression was calculated by the 2−ΔΔCt method.

### 2.5. Western Blot Analysis

Cells were harvested, lysed in ice-cold RIPA buffer (Beyotime, Nantong, China), and centrifuged at 4°C for 15 min at 14,000 rpm. Total protein in sample lysates was quantified using a BCA Protein Assay kit (Thermo Scientific Pierce, Rockford, IL, USA). Equal amounts of protein were separated on 8–12% SDS polyacrylamide gels and transferred to PVDF membranes (Pall, New York, NY, USA). After blocking with 5% milk in TBS containing 0.05% Tween-20 (TBST) for 1 h at 37°C, membranes were incubated for 1 h with anti-BTLA (1 : 1000, ImmunoWay, Plano, TX, USA), anti-IL-10 (1 : 1000, ImmunoWay), anti-CCR7 (1 : 5000, Abcam, Cambridge, MA, USA), or anti-HVEM (1 : 1000, Abcam) primary antibody, washed three times with TBST, incubated with secondary antibody for 40 min, washed three times with TBST, and visualized using Immobilon Western Chemiluminescent HRP Substrate (Millipore, Billerica, MA, USA). GAPDH served as an internal loading control. Densitometric analysis was performed using Image Pro-Plus 6.0 software (Media Cybernetics, Silver Spring, MD, USA). Target protein expression was normalized to that of the reference standard and presented as the ratio to that in the control.

### 2.6. Morphological Observation

For scanning electron microscopy (SEM) [19], cells were cultured on coverslips and prefixed with 2.5% glutaraldehyde for 2 h at 4°C. Postfixation was performed with 1% osmium tetroxide for 1 h at 4°C. After each fixation, the samples were washed twice in PBS containing BSA, dehydrated in graded ethanol, and dried to a critical point in liquid CO2 at 95 bar. Coverslips were then coated with gold by cathodic spraying and examined by SEM (JSM6360LV, JEOL, Japan).

### 2.7. Flow Cytometry Analysis

Harvested cells were stained with fluorescent-conjugated monoclonal antibodies to CD80, CD83, and MHC-II for 30 min at 4°C in the dark and then analyzed using a FACSCalibur flow cytometer with CellQuest software (BD Biosciences, San Jose, CA, USA).

### 2.8. Mixed Lymphocyte Reaction

Mouse lymphocytes were purified with lymphocyte separation liquid (TBD Science, Tianjin, China) according to the manufacturer's protocol. Lymphocytes (5 × 10^4^/well) were cocultured with imDCs inactivated by 25 *μ*g/mL mitomycin C in 96-well plates for 5 days. Pure lymphocyte cultures were used as a control. T cell proliferation was monitored by the addition of 0.5 *μ*Ci/well of 3H-thymidine (Amersham, France) for 18 h, at which point cells were harvested on glass-fiber filter mats and analyzed on a liquid scintillation counter. The stimulation index (SI) for each group was calculated using the average counts per minute (CPM) from triplicate experiments as follows: SI = (CPMtested − CPMcontrol)/(CPMcontrol).

### 2.9. Detection of IFN-*γ*, IL-4, IL-17, and TGF-Beta

Mouse lymphocytes were purified with lymphocyte separation liquid (TBD Science) according to the manufacturer's protocol. Lymphocytes (1 × 10^5^/well) were cocultured with each group imDCs inactivated by 25 *μ*g/mL mitomycin C in 24-well plates for 3 days. Then supernatants were harvested for ELISA assays detecting IFN-*γ*, IL-4, IL-17, and TGF-beta according to the manufacturer's protocol (eBioscience, San Diego, CA, USA). ELISA assays were performed in duplicate and repeated three times.

### 2.10. In Vitro Chemotaxis Assays

The chemotaxis of imDCs to rmCCL19 (R&D Systems, Minneapolis, MN, USA) was examined in 24-well cell culture plates with bare 8.0 *μ*m pore polycarbonate inserts (Corning BV, Schiphol-Rijk, Netherlands). Briefly, 600 *μ*L of RPMI 1640 without or with 100 ng/mL rmCCL19 was added to the lower wells, followed by the addition 100 *μ*L of imDCs (2 × 10^5^ cells) in RPMI 1640 media to the inserts. After a 2 h incubation at 37°C, the inserts were removed and migrated cells were collected by flushing the filter bases and wells with 600 *μ*L RPMI 1640. Migrated cells were counted with a Malassez chamber.

### 2.11. Statistical Analysis

Data were analyzed by Student's *t*-testing and expressed as the mean ± standard deviation (SD). *p* < 0.05 was considered statistically significant.

## 3. Results

### 3.1. CCR7 and/or BTLA Overexpression in imDCs

**Figure 1:**
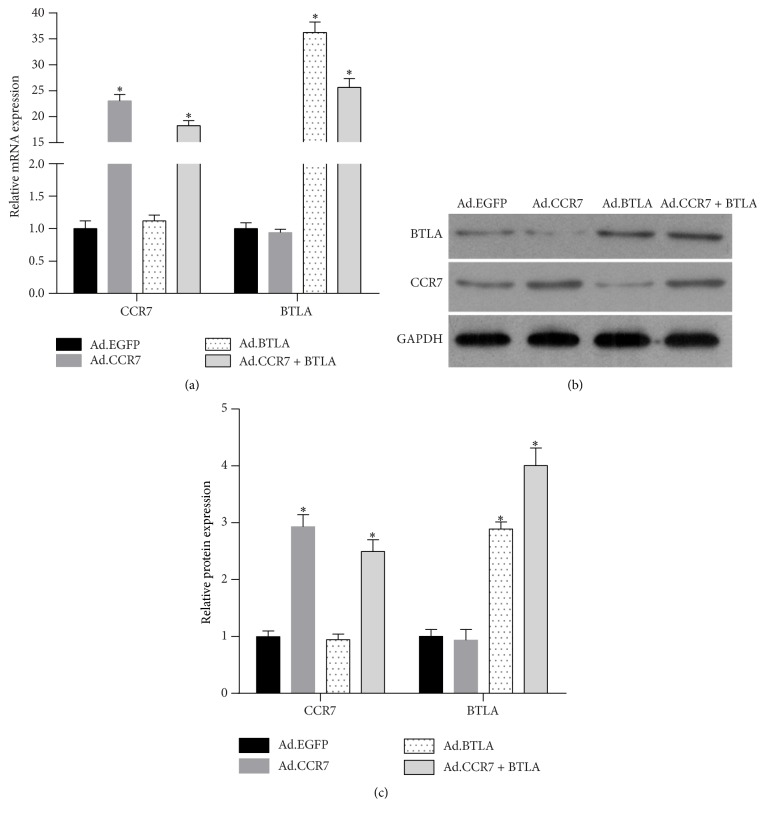
CCR7 and BTLA mRNA and protein expression levels in imDCs infected with Ad.EGFP, Ad.CCR7, Ad.BTLA, or Ad.CCR7 + BTLA. (a) CCR7 and BTLA mRNA expression. (b) Representative image of CCR7 and BTLA protein expression. (c) Relative CCR7 and BTLA protein expression shown as the means ± SD. ^*∗*^*p* < 0.05, when compared to Ad.EGFP group.

Four groups of imDCs were infected with adenovirus as follows: EGFP only control (Ad.EGFP), CCR7-EGFP (Ad.CCR7), BTLA-tdTomato (Ad.BTLA), and Ad.CCR7 and Ad.BTLA (Ad.CCR7 + BTLA). Analysis of CCR7 and BTLA mRNA and protein expression levels confirmed the successful overexpression of each factor when compared to Ad.EGFP-infected controls (Figure 1).

### 3.2. Effect of CCR7 and/or BTLA Overexpression on imDC Cellular Morphology

**Figure 2:**
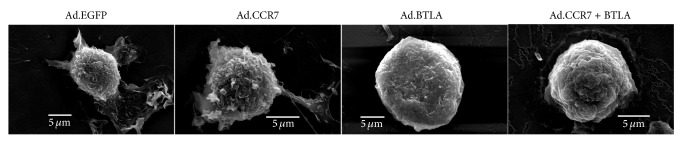
Representative SEM images of imDCs infected with Ad.EGFP, Ad.CCR7, Ad.BTLA, or Ad.CCR7 + BTLA.

Three days after infection with Ad.EGFP, Ad.CCR7, Ad.BTLA, or Ad.CCR7 + BTLA, the cellular morphology of imDCs was examined under SEM (Figure 2). Notably, Ad.EGFP- and Ad.CCR7-infected imDCs exhibited a mature DC phenotype with long, branched protrusions; whereas Ad.BTLA- or Ad.CCR7 + BTLA-infected counterparts displayed a smooth, round morphology with small protrusions typical of imDCs.

### 3.3. Effect of CCR7 and/or BTLA on imDC Cell Surface Antigen Expression

**Figure 3:**
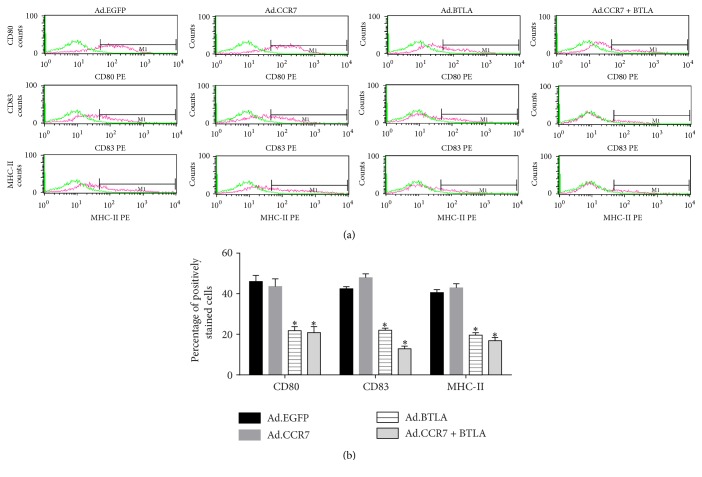
CD80, CD83, and MHC-II surface antigen expression in imDCs infected with Ad.EGFP, Ad.CCR7, Ad.BTLA, or Ad.CCR7 + BTLA. (a) Representative flow cytometry plots. (b) Percentage of positive cells in each group. Data are expressed as the mean ± SD. ^*∗*^*p* < 0.05, when compared to Ad.EGFP group.

The cell surface antigens CD80, CD83, and MHC-II are markers of DC maturation. Thus, imDCs were examined for the presence of these antigens 3 days after infection by flow cytometry (Figure 3). Notably, Ad.EGFP- and Ad.CCR7-infected imDCs expressed high levels of CD80, CD83, and MHC-II, which were less prevalent in Ad.BTLA- and Ad.CCR7 + BTLA-infected counterparts.

### 3.4. Effect of CCR7 and/or BTLA Overexpression on imDC Migration

**Figure 4:**
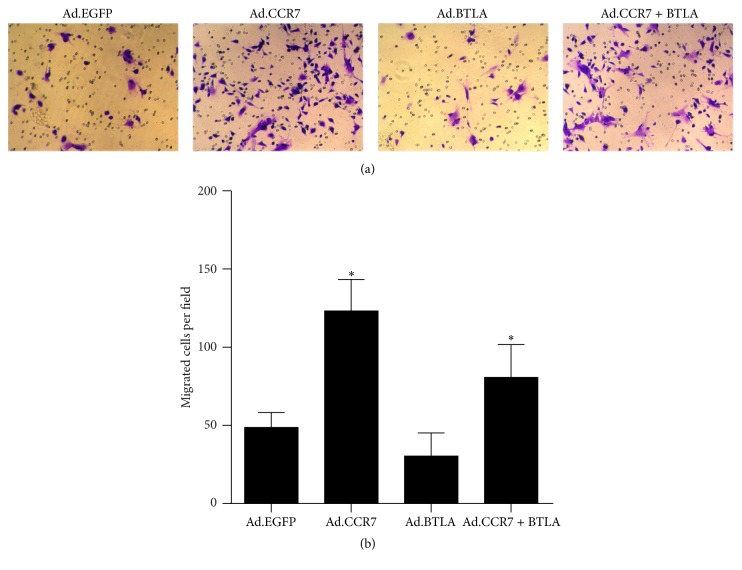
Cell migration was examined in imDCs infected with Ad.EGFP, Ad.CCR7, Ad.BTLA, or Ad.CCR7 + BTLA with in vitro chemotaxis assays. (a) Representative images of migrated cells. (b) Quantification of migrated cells per field expressed as the mean ± SD. ^*∗*^*p* < 0.05, when compared to Ad.EGFP group.

In vitro chemotaxis experiments were performed with cells 3 days after infection to determine the respective effects of CCR7 and BTLA overexpression on imDC migration. As expected, imDCs with BTLA overexpression and EGFP controls showed a weak potential for chemotaxis towards CCL19 (also known as MIP-3*β*), whereas CCR7-overexpressing cells had the strongest migratory ability of the four groups (Figure 4). Notably, imDCs overexpressing both CCR7 and BTLA displayed an intermediate migration phenotype, indicating that BTLA restrained the promigratory effects of CCR7.

### 3.5. Effect of CCR7 and/or BTLA Overexpression on imDC Immune Tolerance

**Figure 5:**
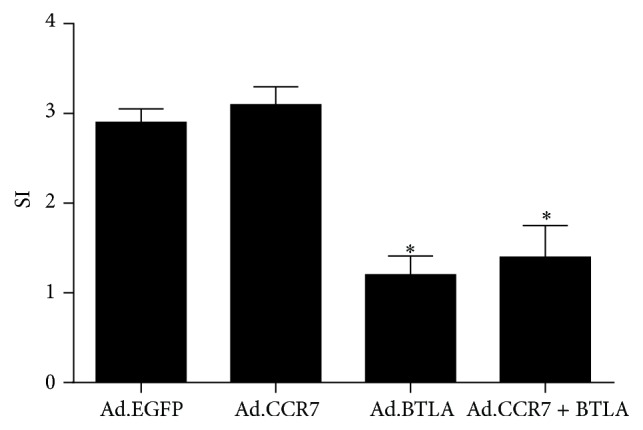
Stimulation index (SI) of imDCs on lymphocyte proliferation in mixed lymphocyte reactions. Ad.EGFP: lymphocyte cocultured with Ad.EGFP-infected imDCs, Ad.CCR7: lymphocyte cocultured with Ad.CCR7-infected imDCs, Ad.BTLA: lymphocyte cocultured with Ad.BTLA-infected imDCs, and Ad.CCR7 + BTLA: lymphocyte cocultured with Ad.CCR7 + BTLA-infected imDCs. Data are expressed as the mean ± SD. ^*∗*^*p* < 0.05, when compared to Ad.EGFP group.

The immune tolerance of imDCs with CCR7 and/or BTLA overexpression was examined in mixed lymphocyte reactions. These studies revealed that Ad.BTLA- or Ad.CCR7 + BTLA-infected imDCs exhibited a potent inhibitory effect on lymphocyte proliferation (SI < 2), which was not observed with Ad.EGFP and Ad.CCR7 counterparts (Figure 5).

### 3.6. Effect of CCR7 and/or BTLA Overexpression on the Expression Level of IFN-*γ*, IL-4, IL-17, and TGF-Beta

**Figure 6:**
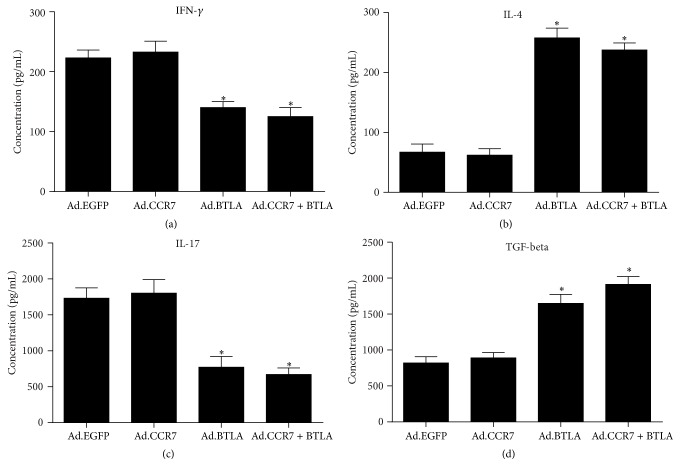
The expression level of IFN-*γ*, IL-4, IL-17, and TGF-beta, secreted by Th1, Th2, Th17, and Treg, respectively. Ad.EGFP: lymphocyte cocultured with Ad.EGFP-infected imDCs, Ad.CCR7: lymphocyte cocultured with Ad.CCR7-infected imDCs, Ad.BTLA: lymphocyte cocultured with Ad.BTLA-infected imDCs, and Ad.CCR7 + BTLA: lymphocyte cocultured with Ad.CCR7 + BTLA-infected imDCs. Data are expressed as the mean ± SD. ^*∗*^*p* < 0.05, when compared to Ad.EGFP group.

To discuss the possible mechanism of that CCR7 and/or BTLA overexpression affecting imDC immune tolerance, we detected the expression level of IFN-*γ*, IL-4, IL-17, and TGF-beta, secreted by T helper 1 (Th1), Th2, Th17, and regulatory T cells (Tregs), respectively. IFN-*γ*, IL-4, IL-17, and TGF-beta expression level of each group lymphocyte cocultured with imDCs overexpressing CCR7 and/or BTLA was examined using ELISA. These studies revealed that Ad.BTLA- or Ad.CCR7 + BTLA-infected imDCs suppressed IFN-*γ* and IL-17 expression and promoted IL-4 and TGF-beta expression compared to Ad.EGFP-infected imDCs (Figures 6(a)–6(d)). However, Ad.CCR7-infected imDCs had no obvious effect on the expression level of IFN-*γ*, IL-4, IL-17, and TGF-beta compared to Ad.EGFP-infected imDCs (Figures 6(a)–6(d)).

## 4. Discussion

A previous study by our group revealed that CCR7-overexpressing imDCs exhibit a strong migratory potential and enhanced ability to stimulate allogeneic T cell proliferation, albeit to a weaker extent than that of mature DCs [13], indicating that CCR7 expression induces a semimature phenotype in imDCs. Thus, the present study sought to identify a method to maintain the beneficial effects of CCR7 expression while preserving the immune tolerance intrinsic to imDCs. For this, we infected imDCs with Ad.CCR7 and/or Ad.BTLA and then examined their respective effects on cell morphology, surface antigen expression, migration, and immune tolerance.

BTLA is an immune inhibitory receptor with widespread effects on T and B cell activity [20]; however, its role in regulating DC maturation is not clear. Normal imDCs have a smooth surface with small protrusions, which develop into long, branched outgrowths in mature counterparts. Notably, our SEM results showed that Ad.BTLA- and Ad.CCR7 + BTLA-infected imDCs exhibit typical imDC morphology in contrast to the more mature appearance of Ad.CCR7-infected cells. This phenotype was further confirmed by flow cytometry analysis for expression of the mature DC surface markers CD80, CD83, and MHC-II [21, 22], which were markedly lower in Ad.BTLA or Ad.CCR7 + BTLA cells when compared to that in Ad.CCR7 and Ad.EGFP controls. Based on these results, we predicted that BTLA overexpression was sufficient to retain the immature status in imDCs.

CCR7 and its ligands are crucial for DC migration [23–25]. The receptor is rapidly upregulated in mature DCs to regulate their migration into the lymphatics via a CCL21 gradient and then to drain lymph nodes in response to CCL19/MIP-3*β* [21]. However, since imDCs are devoid of CCR7 expression, their ability to migrate to secondary lymphatic organs is substantially weakened. We previously reported that imDCs infected with Ad.CCR7 exhibit a strong migratory capacity, but this was accompanied by a heightened immunoresponsiveness that might be detrimental to graft survival [13]. Significantly, in vitro chemotaxis assays in the present study showed that imDCs coinfected with Ad.CCR7 and Ad.BTLA displayed a weaker migratory potential than Ad.CCR7-infected counterparts, but were more adept than Ad.EGFP-infected controls. Moreover, it is generally accepted that imDCs modulate immune tolerance as they are unable to stimulate allogenic T cell proliferation in mixed lymphocyte reactions [5, 6], yet this response was intact in reactions with Ad.CCR7 imDCs, albeit to a lower magnitude than that observed with mature DCs [16]. Interestingly, Ad.CCR7 + BTLA-infected imDCs displayed a tempered response in reaction assays when compared to Ad.CCR7 controls.

Naive CD4^+^ helper T cells (Thp) can develop into at least four types of committed helper T cells, namely, Th1, Th2, Th17, and Treg. In present study, we detected the cytokines expression secreted by Th1, Th2, Th17, and Treg, respectively. The results showed that Ad.CCR7 + BTLA-infected imDCs could suppress IFN-*γ* and IL-17 expression and promoted IL-4 and TGF-beta expression of lymphocyte, indicating an increase of Th2 and Treg cells. The balance of Th1/Th2/Th17/Treg cytokine controls immune response and has been reported to be a key factor in regulating Thp cell function in autoimmune diseases and graft versus host disease [26, 27]. And immune tolerance could be induced via Th2 and Treg cells [28]. So we predicate that Ad.CCR7 + BTLA-infected imDCs enhances immune tolerance via induced Th2 and Treg cells. However, the accurate regulating mechanism needs further study.

In conclusion, our data demonstrate that Ad.CCR7 + BTLA-infected imDCs exhibit a modest capacity for migration and allogeneic T cell stimulation, indicating that these cells serve as an intermediary to maintain immunocompetence, while also retaining the immune tolerance necessary for graft survival. As such, this study provides a basis for further studies on imDCs in immune tolerance, with the goal of developing effective cellular immunotherapies for transplant recipients.
